# F-18-fluoro-2-deoxyglucose positron emission tomography (PET) and PET/computed tomography imaging in primary staging of patients with malignant melanoma: a systematic review

**DOI:** 10.1186/2046-4053-1-62

**Published:** 2012-12-13

**Authors:** Milly A Schröer-Günther, Robert F Wolff, Marie E Westwood, Fülöp J Scheibler, Christoph Schürmann, Brigitta G Baumert, Stefan Sauerland, Jos Kleijnen

**Affiliations:** 1 Institute for Quality and Efficiency in Health Care, Im Mediapark 8, Cologne, 50670, Germany; 2 Kleijnen Systematic Reviews Ltd, Unit 6, Escrick Business Park Riccall Road, Escrick, York, YO19 6FD, UK; 3 School for Public Health and Primary Care (CAPHRI), Maastricht University, Universiteitssingel 40, Maastricht, ER, 6229, the Netherlands; 4 Department of Radiation–Oncology (MAASTRO), GROW (School for Oncology & Developmental Biology), Maastricht University Medical Center, P.O. Box 616, Maastricht, MD, 6200, the Netherlands

**Keywords:** Positron emission tomography, Computed tomography, Staging, Recurrence, Esophageal neoplasms, Systematic review

## Abstract

**Purpose:**

The aim of this systematic review was to systematically assess the potential patient-relevant benefit (primary aim) and diagnostic and prognostic accuracy (secondary aim) of positron emission tomography (PET) and PET/computed tomography (CT) in primary staging of malignant melanoma. This systematic review updates the previous evidence for PET(/CT) in malignant melanoma.

**Materials and methods:**

For the first aim, randomized controlled trials (RCTs) investigating patient-relevant outcomes and comparing PET and PET(/CT) with each other or with conventional imaging were considered. For the secondary aim, a review of reviews was conducted, which was amended by an update search for primary studies. MEDLINE, EMBASE and four databases of the Cochrane Library were searched. The risk of bias was assessed using a modified QUADAS tool.

**Results:**

No RCTs investigating the patient-relevant benefit of PET(/CT) and no prognostic accuracy studies were found. Seventeen diagnostic accuracy studies of varying quality were identified. For patients with American Joint Committee on Cancer (AJCC) stages I and II, sensitivity mostly ranged from 0 to 67%. Specificity ranged from 77 to 100%. For AJCC stages III and IV, sensitivity ranged from 68 to 87% and specificity from 92 to 98%.

**Conclusion:**

There is currently no evidence of a patient-relevant benefit of PET(/CT) in the primary staging of malignant melanoma. RCTs investigating patient-relevant outcomes are therefore required. The diagnostic accuracy of PET(/CT) appears to increase with higher AJCC stages.

## Introduction

Malignant melanoma accounts for about 5% of all skin cancers [1]. Depending mainly on the initial stage of disease, survival after diagnosis can range from only a few months to many years [2]. Once distant metastases have been diagnosed, median survival in untreated patients amounts to 6 to 9 months [3]. Accurate primary staging is therefore essential for developing an appropriate treatment strategy. Conventional techniques for staging include computed tomography (CT), magnetic resonance imaging, skeletal scintigraphy, and conventional X-ray [4].

Positron emission tomography (PET) is a nuclear-medical imaging method that provides information on the function and metabolism of tissue (metabolic imaging) and is used as a stand-alone procedure (PET) or in combination with CT (PET/CT). A number of international societies have concluded that PET(/CT) is useful for detection of metastasis in patients with American Joint Committee on Cancer (AJCC) stages III and IV [2-5]. A current UK guideline states that there is good evidence against the use of PET(/CT) for detection of metastasis in patients with AJCC stages I and II [6].

This systematic review formed part of a health technology assessment report that will be used to guide national policy on the reimbursement of PET(/CT) in Germany.

The full protocol and report are available on the website of the responsible health technology assessment agency [7,8], whose tasks and methodological approach are described in its paper on general methods [7,8]. The main aim of this systematic review was to assess the potential patient-relevant benefit of PET(/CT) in primary staging of malignant melanoma. Our secondary aim was to assess the diagnostic and prognostic accuracy of PET(/CT) in the same indication (detection of regional lymph node metastases and/or diagnostic accuracy for detection of distant metastases).

## Materials and methods

### Search strategy and study selection

For our primary aim (that is, assessment of patient-relevant benefit) we searched for relevant randomized controlled trials (RCTs) investigating at least one predefined patient-relevant outcome (see below). In this context, the term ‘patient-relevant’ refers to how a patient feels, functions or survives. If insufficient evidence was available to answer the primary question, an assessment of the diagnostic and prognostic accuracy of PET(/CT) was performed (secondary aim). For this purpose, a review of reviews was conducted, and where appropriate, supplemented with additional recent primary studies identified by our own update search.

Primary studies were searched for in MEDLINE (1948 to January 2011) and EMBASE (1980 to January 2011) via Ovid, and in the Cochrane Central Register of Controlled Trials. The Cochrane Database of Systematic Reviews, the Database of Abstracts of Reviews of Effects, and the Health Technology Assessment Database were screened to identify systematic reviews. In addition, reference lists of retrieved articles and conference proceedings were searched by hand. Databases of guideline developers were also searched to identify further systematic reviews. Finally, web-based clinical trial registries and trial results databases were screened. The search strategy included bibliographic index terms on melanoma and PET. The full search strategy, which was developed by one information specialist and checked by another, has been described elsewhere [7,8]. Two reviewers independently screened titles and abstracts of the retrieved citations to identify potentially eligible primary and secondary publications. The full texts of these articles were obtained and independently evaluated by the same two reviewers applying the full set of inclusion and exclusion criteria. Disagreements were resolved by consensus.

### Eligibility criteria

For our primary aim, RCTs with the following characteristics were included. First, the RCT investigated patients with malignant melanoma. Second, the trial evaluated at least one of the following predefined patient-relevant outcomes: health-related quality of life, melanoma-related mortality, all-cause mortality, and other adverse events. Third, in the RCT either PET and/or PET/CT were compared with each other or with another diagnostic test (for example, CT), or PET or PET/CT was used to identify patients and assign them to different treatment regimens based on the test result (for example, enrichment design). Finally, there was a full-text document available (no language restrictions).

For the secondary aim, systematic reviews identified in the literature search were evaluated using Oxman and Guyatt’s quality tool [9,10]. Eligible systematic reviews had to achieve five or more of seven possible points in order to be included in our review. In addition, we conducted an update search for primary studies published up to January 2011 to cover the period not considered by the systematic reviews.

With regard to diagnostic and prognostic accuracy, we extracted and analyzed data from primary studies (identified either by the published high-quality systematic reviews or by our literature search) if the following criteria were fulfilled:

prospective design; patient-based analysis; index test (PET or PET/CT); valid reference standard (histopathology, clinical follow-up >6 months, or a combination of the two) – if histological analyses were not possible (for example, for M-staging), we also accepted studies that only applied clinical-radiologic follow-up; number of patients (at least 10); sufficient data for calculation of 2×2 tables; and full-text document available.

Where the number of studies that investigated a direct comparison of PET or PET/CT with other diagnostic procedures was insufficient, we also included verification of only positive testers design studies and discordance studies. In the verification of only positive testers design, the reference standard is applied only to those patients in whom a suspicious lesion was found by one or both of the index tests. In the discordance design, the reference standard is applied only to those patients in whom one test, but not both tests, was positive (that is, where there is a discordant result).

### Data extraction

The individual steps of the data extraction and risk-of-bias assessment procedures were conducted by one reviewer and checked by another; disagreements were resolved by consensus. As no RCTs and prognostic accuracy studies were identified, no further details of the planned data extraction and risk-of-bias assessment are provided here.

Using standardized tables, information was extracted from each included diagnostic study on: baseline characteristics of the study participants; characteristics of the index test and reference standard; data for constructing 2×2 contingency tables of diagnostic and prognostic accuracy (that is, numbers of true-positive, false-negative, false-positive and true-negative test results); reported sensitivities and specificities of PET(/CT); and risk-of-bias items (see below).

### Assessment of risk of bias

The risk of bias in diagnostic accuracy studies identified in the update search was evaluated using a modified version of QUADAS, an evidence-based tool recommended for assessing the methodological quality of test accuracy studies [11]. We modified this tool because, in our opinion, some QUADAS items referred more to external validity than to internal validity. Since our review was undertaken, a revised version of QUADAS (QUADAS-2) has been released [12]. QUADAS-2 more closely resembles the approach and structure of the Cochrane risk-of-bias tool. Furthermore, it aims to produce an assessment of the risk of bias by methodological domain (participant selection, index test, reference standard, and flow of participants through the study), rather than the more general overall risk of bias.

The following criteria were assessed: item 1, application of a reference standard likely to correctly classify the target condition; item 2, observance of an appropriate time period between application of the reference standard and the index test; item 3, independence of the index test from the reference standard; items 4 to 6, avoidance of partial verification bias, differential verification bias, and incorporation bias; item 7, interpretation of the reference standard without knowledge of the results of the index text; item 8, performance of an intention-to-diagnose analysis; item 9, avoidance of selective reporting; and item 10, no identification of other aspects contributing to a risk of bias.

The QUADAS tool does not recommend the use of algorithms to derive overall ratings of study quality. However, the revised version of the QUADAS tool (QUADAS-2) allows for an overall rating of ‘low risk of bias, where all criteria are met’ [12]. For this evaluation we developed topic-specific scoring guidance. In particular, we modified the requirement for all criteria to be met, to allow for the fact it was not possible for studies to meet item 5 (differential verification bias). The studies were then categorized as follows. If one of the items was evaluated with ‘no’ (except item 5), the study was rated as having a high risk of bias. If at least two of the items were rated as ‘unclear’, the study was also rated as having a high risk of bias – items 7, 8 and 10 were exceptions; here at least two items (plus an additional one from items 1 to 6 or 9) had to be rated as ‘unclear’ in order to conclude a high risk of bias.

The risk of bias in the diagnostic accuracy studies identified by the systematic reviews was classified according to the ratings given in the respective reviews.

### Data analysis

Sensitivity and specificity were calculated from contingency tables as reported in or derived from the studies. The 95% confidence intervals were computed using the Clopper and Pearson method [13]. Owing to great clinical heterogeneity, bivariate analyses did not seem appropriate. The meta-analyses of the diagnostic accuracy values from the included studies are presented in forest plots according to analyses of all patients with AJCC stages I to IV and of two subgroups (AJCC stages I and II, and AJCC stages III and IV).

## Results

### Literature search

**Figure 1:**
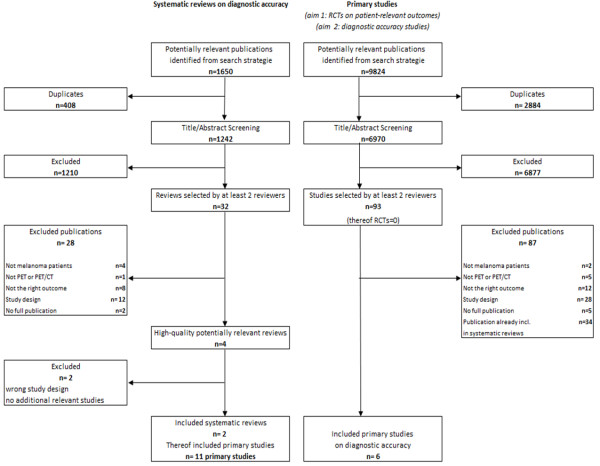
Flow chart of study.

The search for primary studies (that is, the search for RCTs investigating the patient-relevant benefit of PET(/CT) as well as the update search for diagnostic and prognostic accuracy studies) retrieved 9,824 references. However, no relevant RCT on the primary aim of our review was identified (Figure 1).

Concerning the secondary aim, four high-quality potentially relevant systematic reviews on the diagnostic accuracy of PET(/CT) in melanoma [14-17] were retrieved from 1,650 citations. After extraction of the study information, the systematic reviews by Mijnhout and colleagues [16] and Xing and colleagues [17] were excluded from our review. Additional studies investigated by Mijnhout and colleagues and by Xing and colleagues that were not already included through Jiménez-Requena and colleagues [14] or Krug and colleagues [15] did not fulfill our inclusion criteria due to retrospective study designs, due to the reporting of solely nonpatient-based analyses, or because 2×2 tables could not be reconstructed. However, both of the excluded high-quality reviews are addressed in the discussion section of this paper. We did not find any discordance studies or studies using a verification of only positive testers design.

Our review of reviews was ultimately based on primary studies included in two systematic reviews [14,15].

Eleven primary studies included in these reviews met our inclusion criteria.

The searches conducted in the two included reviews ended in March 2007. Our update search therefore covered the period from March 2007 to January 2011 and identified six further relevant studies. In total we therefore included 17 primary studies on diagnostic accuracy in our review. No relevant prognostic study was found.

### Study characteristics

The characteristics of the 17 diagnostic accuracy studies (1,155 patients) are presented in Additional file 1. Twelve studies investigated PET and five studies investigated PET/CT; the latter studies were exclusively identified by our update search. All included studies used F-18-fluoro-2-deoxyglucose as a tracer.

The age of patients ranged from 18 to 89 years. The mean percentage of males per study was 51.0% and the mean number of participants was 68 (range 17 to 251). The studies were published between 1998 and 2010.

Six studies included patients with primary tumors in AJCC stages I and II [18-23]. Three studies analyzed patients with AJCC stages III and IV: patients with AJCC stages II and III [24], patients with AJCC stages II to IV [25], and patients with AJCC stages I to III [26]. For three further studies, information on AJCC stages was missing [27-29].

### Risk of bias

The risk of bias varied across studies. Both systematic reviews used different assessment tools. Jiménez-Requena and colleagues modified items from previous systematic reviews on PET [30-33]. These items covered seven dimensions: ‘description of study design, description of the study population, indications leading to F-18-fluoro-2-deoxyglucose PET use, technical and image interpretation issues, final confirmation, sensitivity and specificity data, and change in management information’ [14]. A score >70% was defined as high quality (low risk of bias); six of the 10 studies included in our review were defined as having a low risk of bias by Jiménez-Requena and colleagues [18-20,24-26].

Krug and colleagues assessed the risk of bias using the QUADAS tool [11]. Detailed information was provided on the potential sources of bias and the proportion of affected studies. These sources were, among others: the inclusion of an inappropriate range of patients, the insufficient description of inclusion and exclusion criteria, insufficient information on primary tumors, the lack of an independent interpretation of the index test and reference standard, and insufficient explanation of withdrawals. However, only a QUADAS sum score was provided for individual studies, which does not discriminate between what could be a wide variety of quality issues (different sources of bias and reporting and applicability issues) and in addition does not distinguish between internal and external validity. Furthermore, no categorization of studies into those with a high risk and those with a low risk of bias was performed. The QUADAS sum score was developed by Krug and colleagues, which is not recommended by the authors of QUADAS. However, one study fulfilled all QUADAS criteria [20].

The seven studies included in both reviews were rated similarly [18-20,22,24,26,27], independent of the assessors. All studies except the study by Rinne and colleagues [22] were classified as having a low risk of bias.

Of the six studies published after March 2007 and identified in the update search [23,28,34-37], two had a high risk of bias [28,35]. The main weaknesses of these two studies were: the time period between the reference test and the index test was inappropriate or unclear (Table 1, item 2), incorporation bias was evident (item 6), the results of the reference test were interpreted with knowledge of the results of the index test or the corresponding information was missing (item 7), or no intention-to-diagnose analysis was performed (item 8).

**Table 1:** Risk-of-bias assessment of studies published after March 2007

**Study**	**Item 1**	**Item 2**	**Item 3**	**Item 4**	**Item 5**^**a**^	**Item 6**	**Item 7**	**Item 8**	**Item 9**	**Item 10**	**Risk of bias**
Maubec and colleagues [28]	Yes	Unclear	Yes	Yes	No	Yes	Unclear	Yes	Yes	Unclear	Low
Strobel and colleagues [36]	No	Unclear	Yes	Yes	No	No	Unclear	Unclear	Yes	Unclear	High
Singh and colleagues [23]	Yes	Unclear	Yes	Yes	No	Yes	No	Unclear	Yes	Unclear	High
Bastiaannet and colleagues [35]	Yes	Unclear	Yes	Yes	No	Yes	Unclear	Unclear	Yes	Unclear	Low
Veit-Haibach and colleagues [37]	Yes	Yes	Yes	Yes	No	Unclear	Yes	No	Yes	Unclear	High
Aukema and colleagues [34]	Unclear	Unclear	Yes	Yes	No	Unclear	Unclear	Yes	Yes	Unclear	High

Item 1, reference standard likely to correctly classify the target condition appropriately; item 2, time period between reference standard and index test appropriate; item 3, interdependence of test appropriate; item 4, partial verification avoided; item 5, differential verification bias; item 6, incorporation bias; item 7, reference standard results interpreted without knowledge of the results of the index test; item 8, intention-to-diagnose analysis performed; item 9, Free from selective reporting; item 10, no other aspects. Yes, low risk of bias; no, high risk of bias; unclear, information for assessment of the item is missing.

^a^Positron emission tomography (PET) studies had a differential verification bias because PET nonresponders were only followed up, instead of undergoing a biopsy. Item 5 was not considered in the risk-of-bias assessment.

## Analyses

### Diagnostic accuracy

The results for diagnostic accuracy are displayed in Table 2. We did not pool data in meta-analyses because of differences in indications (N-staging or M-staging, or a mix of both), reference standards and index tests. The sensitivity of PET(/CT) of all included studies ranged from 0 to 100%; specificity ranged from 18 to 100%. Eleven analyses (nine studies) on the detection of regional lymph node metastases (N-staging) were included. Seven analyses were based on PET and four on PET/CT. They demonstrated a sensitivity and specificity for PET(/CT) of 0% (specificity 80.6%) to 100% (specificity 77%) and 18% (sensitivity 40%) to 100% (sensitivity 8 to 38%), respectively [18,19,21,23,25-27,34,37]. Seven analyses were based on PET and four on PET/CT.

**Table 2:** Results of included studies

**Study**	**Indication and index test**		**TP**	**FN**	**FP**	**TN**	**Sensitivity (%) (95% CI)**	**Specificity (%) (95% CI)**
Rinne and colleagues [22]	N-staging and M-staging	PET	4	0	11	37	100 (40 to 100)	77 (63 to 88)
Crippa and colleagues [27]	N-staging	PET	17	2	3	16	90 (66.9 to 98.7)^b^	84 (60.4 to 96.6)^b^
Eigtved and colleagues [24]	M-staging	PET	28	1	4	5	97 (82 to 100)	56 (21 to 86)
Klein and colleagues [21]	N-staging	PET	2	1	0	14	67 (9.4 to 99.2)^b^	100 (76.8 to 100)^b^
Acland and colleagues [18]	N-staging	PET	0^a^	14^a^	7^a^	29^a^	0 (0 to 23.2)^b^	80,6^b^ (64.0 to 91.8)^b^
Belhocine and colleagues [19]	N-staging	PET	1^a^	5^a^	1^a^	14^b^	16.7^b^ (0.4 to 64.1)^b^	93.3^b^ (68.1 to 99.8)^b^
Reinhardt and colleagues [29]	M-staging	PET	23	2	1	41	92 (74.0 to 99.0)^b^	98 (87.4 to 99.9)^b^
Hafner and colleagues [25]	M-staging	PET	0	0	2	98	–	98 d (93.0 to 99.8)^d^
	N-staging	PET	2	24	0	74	8 (0.9 to 25.1)^b^	100 (95.1 to 100)^b^
Fink and colleagues [20]	N-staging	PET	1	7	0	40	13 (0 to 53)	100 (91 to 100)
Vereecken and colleagues [26]	N-staging	PET	4^a^	6^a^	27^a^	6^a^	40.0^b^ (12.2 to 73.8)^b^	18.2^b^ (7.0 to 35.5)^b^
Brady and colleagues [45]	N-staging and M-staging	PET	30	14	5	54	68 (52 to 81)	92 (81 to 97)
Maubec and colleagues [28]	N-staging and M-staging	PET/CT	1^b^	5^b^	5	14	17 (0.4^b^ to 64)	74 (49 to 91)
Strobel and colleagues [36]	N-staging and M-staging	PET/CT	45	8	3	68	85 (72.4 to 93.3)^b^	96 (88.1 to 99.1)^b^
		PET/CT and separate CT	52	1	4	67	98 (89,9 to 100)^b^	94 (86.2 to 98.4)^b^
Singh and colleagues [23]	N-staging	PET/CT	2	12	2	36	14.3 (2.5 to 44) ^b^	94.7 (81 to 99)
Bastiaannet and colleagues [35]	M-staging	PET	68	11	11	161	86.1^b^ (76.5 to 92.8)^b^	93.6^b^ (88.8 to 96.8)^b^
		CT	61	17	11	162^b^	78.2^b^ (67.4 to 86.8)^b^	93.6^b^ (88.9 to 96.8)^b^
Veit-Haibach and colleagues [37]	N-staging	PET/CT	5^b^	8	0	43^b^	38.5 (14 to 68)	100 (92 to 100)
		PET	5^b^	8	0	43^b^	38.5 (14 to 68)	100 (92 to 100)
		CT	3^b,c^	10	0	43^b,c^	23.1 (5 to 53)	100 (92 to 100)
	M-staging	PET/CT	5^b^	7	3	41^b^	41.7 (15 to 72)	93.2 (81 to 99)
		PET	4^b^	8	4	40 ^b^	33.3 (9 to 65)	90.9 (78 to 97)
		CT	3^b,c^	9	3	41^b,c^	25.0 (5 to 57)	93.2 (81 to 99)
Aukema and colleagues [34]	N-staging	PET/CT	26	4	1	39	86.7^b^ (69.3 to 96.2)^b^	97.5^b^ (86.8 to 99.9)^b^

CI, confidence interval; CT, computed tomography; FN, false-negative; FP, false-positive; PET, positron emission tomography; TN, true-negative; TP, true-positive;

^a^Data were extracted from primary study.

^b^Our own calculation.

^c^The combination of TP and TN is reported in Table 3 of the study publication. Our own calculation of these values was possible because of the additional information provided in the publication.

Six analyses on the detection of distant metastases (M-staging) were performed in five studies [24,25,29,35,37]. Five analyses were based on PET and one on PET/CT. The sensitivity of PET(/CT) ranged from 33% (specificity 90.3%) to 97% (specificity 56%) and specificity from 56% (sensitivity 97%) to 98% (sensitivity 92%) [24,25,29,35,37].

Four studies reported contingency tables for both indications (N-staging and M-staging) [20,22,28,36].

In two studies, differences in diagnostic accuracy between imaging procedures were tested for statistical significance [35,37]. Veit-Haibach and colleagues analyzed PET versus PET/CT and PET versus CT [37]. Sensitivity was low for all technologies whereas specificity was high. No statistically significant differences were detected between the technologies.

Analyses by Bastiaannet and colleagues showed that PET and CT performed similarly regarding the detection of liver, lung and abdominal metastases (*P* = 0.81, *P* = 0.16 and *P* = 0.62, respectively, no estimates and confidence intervals provided) [35].

### Subgroup analysis

**Figure 2:**
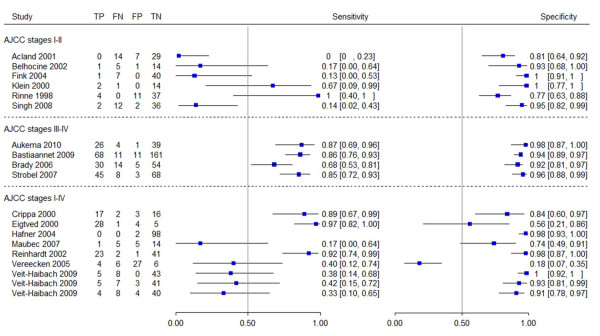
Forest plots of diagnostic accuracy.

In patients with AJCC stages I and II, the sensitivity of PET(/CT) ranged from 0% (specificity 81%) to 67% (specificity 100%) (Figure 2). In four studies of this subgroup, the sensitivity of PET(/CT) ranged from 0% (specificity 81%) to 17% (specificity 93%), while in the other two studies the sensitivity was 67% (specificity 100%) and 100% (specificity 100%). The specificity of PET(/CT) in this subgroup ranged between 77% (sensitivity 100%) and 100% (sensitivity 13 or 67%). Three studies investigated AJCC stages III and IV; in this second subgroup, the sensitivity of PET(/CT) ranged from 68% (specificity 92%) to 87% (specificity 98%) (specificity 92% (sensitivity 68%) to 98% (sensitivity 87%)).

In the third group, studies including patients in all stages (AJCC stages I to IV) were compared. The sensitivity of PET(/CT) ranged from 17% (specificity 74%) to 97% (specificity 56%) and specificity ranged from 18% (sensitivity 40%) to 100% (sensitivity 38%).

Veit-Haibach and colleagues analyzed M-staging and N-staging for PET(/CT) [37]. The 2×2 tables for PET(/CT) for N-staging showed identical results. Only three 2×2 tables are therefore presented in Figure 2. Sensitivity ranged from 17% (specificity 74%) to 97% (specificity 56%). Sensitivity could not be calculated for Hafner and colleagues as no true-positive and false-negative cases were detected by PET [25]. In six studies, specificity ranged from 56% (sensitivity 97%) to 100% (sensitivity 38%), except for Vereecken and colleagues [26], with a specificity of 18% (sensitivity 40%).

## Discussion

Despite a comprehensive search, no RCT investigating the potential patient-relevant benefit of PET(/CT) in primary staging of malignant melanoma was identified. Only diagnostic accuracy studies were found, which were retrieved from two previous systematic reviews and from an update search.

For N-staging and M-staging, the results of the individual studies varied widely in their estimates of sensitivity and specificity. Potential sources of heterogeneity included differences in the risk of bias, in the reference tests and index tests applied and in the spectrum of patients investigated. Stratification by disease stage indicated that the diagnostic accuracy of PET(/CT) varied with AJCC stage. PET(/CT) performed better in patients with AJCC stages III and IV than in those with lower AJCC stages. However, higher sensitivity and specificity of PET(/CT) in AJCC stages III and IV do not necessarily imply that there is a patient-relevant benefit of PET(/CT) in this subgroup. The aim of diagnostic accuracy studies is only to explain how well a new diagnostic test agrees with the reference standard. One cannot draw conclusions from diagnostic accuracy studies as to how variations in the test strategy may ultimately affect a patient’s outcomes. Likewise, robust conclusions cannot be drawn from nonrandomized studies as the potential risk of bias is higher than in randomized ones [38-40]. Moreover, diagnostic accuracy studies are unable to answer effectiveness questions. Owing to the lack of direct comparisons in our review, there is no evidence that the diagnostic accuracy of PET(/CT) is better than conventional imaging.

Our search identified four high-quality systematic reviews investigating diagnostic accuracy [14-17], of which two were included in the actual assessment.

All four reviews conducted meta-analyses: Xing and colleagues [17] and Jiménez-Requena and colleagues [14] presented separate analyses for N-staging and M-staging, whereas Krug and colleagues [15] and Mijnhout and colleagues [16] presented combined analyses. However, in most cases Jiménez-Requena and colleagues refrained from pooling data due to heterogeneity. In contrast, we refrained from conducting any meta-analyses at all because, in our view, the studies were too heterogeneous; for example, concerning patient characteristics (AJCC stage, age, primary cancer site, and so forth) or the use of different index or reference tests (for example, newer studies applied PET/CT whereas older ones applied PET).

For information purposes, we present the results of the meta-analyses or ranges for individual studies in Table 3. However, we emphasize that the results lack comparability due to various factors.

**Table 3:** Results of meta-analyses on diagnostic accuracy

		**Sensitivity (%) (95% CI)**^**a**^	**Specificity (%)(95% CI)**^**a**^
**N-staging**			
Our review	PET (analyzed by patients)	**0 to 90**^e^	**18.2 to 100**^e^
	PET/CT (analyzed by patients)	**14 to 87**^e^	**95 to 100**^e^
Xing and colleagues [17]	PET (analyzed by patients and lesions; mixed)^b^	30 (12 to 55)	96 (87 to 99)
	PET/CT (analyzed by patients and lesions; mixed)^b^	11 (1 to 50)	97 (97 to 100)
Jimenéz-Requena and colleagues [14]	PET (analyzed by lymph nodes)^b^	**–**^c^	99 (97 to 99)
	PET (analyzed by patients)	–^c^	–^c^
	PET (analyzed by areas)^b^	–^c^	–^c^
**M-staging**			
Our review	PET (analyzed by patients)	**33 to 97**^**e**^	**56 to 98**^**e**^
	PET/CT (analyzed by patients)	**42 (15 to 72)**^**d**^	**93 (81 to 99)**^**d**^
Xing and colleagues [17]	PET (analyzed by patients and lesions; mixed)^b^	74 (51 to 88)	75 (45 to 91)
	PET/CT (analyzed by patients and lesions; mixed)^b^	80 (53 to 93)	87 (54 to 97)
Jimenéz-Requena and colleagues [14]	PET (analyzed by patients)	–^c^	–^c^
	PET (analyzed by lesions)^b^	**–**^c^	**–**^c^
	PET (analyzed by areas)^b^	86 (82 to 89)	**–**^c^
	PET (analyzed by Scans)^b^	**–**^c^	86 (77 to 92)
**N-staging and M-staging**			
Our review	PET(analyzed by patients)	**0 to 100**^e^	**18 to 100**^e^
	PET/CT (analyzed by patients)	**17 to 85**^e^	**74 to 96**^e^
Krug and colleagues [15]	PET (analyzed by patients, lesions and scans; mixed)^b^	83 (81 to 84)	85 (83 to 87)
Mijnhout and colleagues [16]	PET (analyzed by patients, lesions and scans; mixed)^b^	78 (70 to 84)	88 (82 to 92)

CT, computed tomography; PET, positron emission tomography. ^a^Pooled estimate with 95% confidence interval (CI), except for: Xing and colleagues, specification of median with credible intervals; Jiménez-Requena and colleagues, specification of mean values with CI; our review, specification of ranges. ^b^The results of these nonpatient-based analyses are not relevant for our review. ^c^Authors reported that quantitative synthesis was not possible due to heterogeneity. ^d^Data based on only one study. ^e^Since the data were too heterogeneous, a quantitative synthesis of the results was not possible. Therefore a range of data is provided. Data of our systematic review is highlighted in bold font face.

For example, all four of the other reviews included both prospective and retrospective studies whereas we only included prospective ones. The quality of studies with a retrospective design is limited due to factors such as spectrum bias, attrition bias, unclear blinding, the often lacking standardised implementation of the index and reference test, and especially the lack of the prospective definition of cutoff points [41]. For future reviews we would suggest performing a comparison of results between study types (prospective vs. retrospective); for example, in the form of sensitivity analyses.

A further reason for the noncomparability of results is the fact that the analyses in the other reviews were largely not exclusively patient-based, but lesion-based. From a statistical point of view, a lesion-based analysis overestimates the precision of results. In our opinion, if not properly accounted for, this type of analysis also ignores the fact that observations on lesions of a given patient are not independent, which may lead to bias.

As already recommended in the literature [42], we suggest that studies on PET imaging report a patient-based analysis as the primary analysis; other types of analyses should always be adjusted for patients or should only be presented as supplementary information.

Furthermore, when examining the diagnostic accuracy of PET in N-staging, we could not reproduce some of the results presented by Xing and colleagues [17], who, for example, reported a specificity of 100% for the studies by Kokoska and colleagues and Longo and colleagues [43,44]. However, specificity in these studies was not reported and could not be calculated; therefore, despite the high-quality rating according to the Oxman and Guyatt tool, we have some doubts about the validity of the analysis performed by Xing and colleagues.

### Assessment of risk of bias in primary studies

Different reference standards will be employed in patients with positive and negative PET scan results. Most metastases will be confirmed histologically, while apparently disease-free patients will remain under regular clinical observation. This issue should be listed as a possible source of differential verification bias (item 5) for all studies; the item’s possible impact is then a matter of judgment – scoring this item negatively does not necessarily mean that the whole study was biased. We judged that histology results and follow-up results will often have similarly high validity, so that this item was essentially not used to decide about the validity of included studies.

### Limitations

One of the major limitations of this systematic review is the low quality of reporting in the studies considered. Studies were often described inadequately. In some studies it was not possible to determine whether they had a retrospective or a prospective design. Furthermore, in some cases it was not clear which cutoff values were used and when the index test PET(/CT) or the reference test was applied.

We performed a review of reviews. Results of risk-of bias assessment were therefore taken from included reviews. A comparison of the results of the risk-of-bias assessment was hampered by the fact that different assessment tools were used and by inadequacies such as a lack of distinction between internal and external validity or a lack of categorization of studies into those with a high or a low risk of bias. In accordance with the authors of the reviews, we also concluded that many studies failed to provide sufficient details to adequately assess risk-of-bias items.

## Conclusions

There is currently no evidence for a patient-relevant benefit of PET(/CT) in primary staging of malignant melanoma, indicating that it may be too early for broad clinical use of this technology. In future, stage-adapted RCTs investigating patient-relevant outcomes are needed to determine whether PET(/CT) actually has a benefit for patients.

The diagnostic accuracy of PET(/CT) appears to increase with higher AJCC stages. However, regardless of the indication (N-staging or M-staging), the ranges for sensitivity and specificity were wide. In addition, the 17 studies on diagnostic accuracy were heterogeneous, some were small and many showed methodological deficiencies. Future diagnostic accuracy studies should be prospective, of better quality, and better reported.

## Abbreviations

AJCC: American Joint Committee on Cancer; CT: Computed tomography; PET: Positron emission tomography; RCT: Randomized controlled trial.

## Competing interests

The authors declare that they have no competing interests.

## Authors’ contributions

MAS-G wrote the main part of the manuscript, made substantial contributions to conception design and drafting, and was involved in acquisition and interpretation of data. RFW was involved in collection and interpretation of data, was involved in writing and drafting the manuscript, and gave final approval for the manuscript. MEW was involved in collection and interpretation of data, was involved in writing and drafting the manuscript, and gave final approval for the manuscript. FJS made substantial contributions to the conception and design, was involved in interpretation of data and was involved with final approval of the manuscript. CS performed all statistical analyses, and gave final approval for the manuscript. BGB made substantial contributions to the clinical part of the manuscript, and gave final approval for the manuscript. SS made substantial contributions to conception and design, was involved in interpretation of data and drafting the manuscript, and gave final approval for the manuscript. JK made substantial contributions to conception and design, was involved in interpretation of data, was involved in drafting the manuscript, and gave final approval for the manuscript.

## Supplementary Material

Additional file 1 **Characteristics of included studies.** Table provides all informations concerning characteristics of included studies. Click here for file

## References

[B1] National Collaborating Centre for Cancer Guidance on Cancer Services: Improving Outcomes for People with Skin Tumours including Melanoma; The Manual, Vol. 2009 2006 National Institute for Health and Clinical Excellence, London

[B2] Garbe C Peris K Hauschild A Saiag P Midddleton M Spatz A Grob JJ Malvehy J Diagnosis and treatment of melanoma: European consensus-based interdisciplinary guideline Eur J Cancer Clin Oncol 2010 46 270 283 10.1016/j.ejca.2009.10.032 19959353

[B3] Diagnosis and Treatment of Melanoma: European Consensus-based Interdisciplinary Guideline http://www.ecco-org.eu/binarydata.aspx?type=doc/diagnosis_and_treatment_of_melanoma.pdf 10.1016/j.ejca.2009.10.032 19959353

[B4] Dummer R Hauschild A Pentheroudakis G Cutaneous malignant melanoma: ESMO clinical recommendations for diagnosis, treatment and follow-up Ann Oncol 2009 20 Suppl 4 129 131 19454433 10.1093/annonc/mdp152

[B5] Saiag P Bosquet L Guillot B Verola O Avril MF Bailly C Cupissol D Dalac S Danino A Dréno B Grob JJ Leccia MT Renaud-Vilmer C Négrier S de Dermatologie SF Management of adult patients with cutaneous melanoma without distant metastasis: 2005 update of the French standards, options and recommendations guidelines; summary report Eur J Dermatol 2007 17 325 331 17540641 10.1684/ejd.2007.0209

[B6] Marsden JR Newton-Bishop JA Burrows L Cook M Corrie PG Cox NH Gore ME Lorigan P MacKie R Nathan P Peach H Powell B Walker C British Association of Dermatologists Clinical Standards Unit Revised U.K. guidelines for the management of cutaneous melanoma Br J Dermatol 2010 163 238 256 10.1111/j.1365-2133.2010.09883.x 20608932

[B7] Allgemeine Methoden: Version 4.0 https://www.iqwig.de/download/IQWiG_Entwurf_Methoden_Version_4-0.pdf

[B8] Positronenemissionstomographie (PET und PET/CT) bei malignen Melanom https://www.iqwig.de/download/D06-01F_Abschlussbericht_PET_und_PET-CT_bei_malignem_Melanom.pdf

[B9] Oxman AD Guyatt GH Validation of an index of the quality of review articles J Clin Epidemiol 1991 44 1271 1278 10.1016/0895-4356(91)90160-B 1834807

[B10] Oxman AD Guyatt GH Singer J Goldsmith CH Hutchison BG Milner RA Streiner DL Agreement among reviewers of review articles J Clin Epidemiol 1991 44 91 98 1824710 10.1016/0895-4356(91)90205-n

[B11] Whiting P Rutjes AW Reitsma JB Bossuyt PM Kleijnen J The development of QUADAS: a tool for the quality assessment of studies of diagnostic accuracy included in systematic reviews BMC Med Res Methodol 2003 3 25 10.1186/1471-2288-3-25 14606960 PMC305345

[B12] Whiting PF Rutjes AW Westwood ME Mallett S Deeks JJ Reitsma JB Leeflang MM Sterne JA Bossuyt PM QUADAS-2: a revised tool for the quality assessment of diagnostic accuracy studies Ann Intern Med 2011 155 529 536 22007046 10.7326/0003-4819-155-8-201110180-00009

[B13] Clopper CJ Pearson ES The use of confidence or fiducial limits illustrated in the case of the binominal Biometrika 1934 26 404 413 10.1093/biomet/26.4.404

[B14] Jiménez-Requena F Delgado-Bolton RC Fernandez-Perez C Gambhir SS Schwimmer J Perez-Vazquez JM Carreras-Delgado JL Meta-analysis of the performance of 18F-FDG PET in cutaneous melanoma Eur J Nucl Med Mol Imaging 2010 37 284 300 10.1007/s00259-009-1224-8 19727717 PMC2886141

[B15] Krug B Crott R Lonneux M Baurain JF Pirson AS Vander Borght T Role of PET in the initial staging of cutaneous malignant melanoma: systematic review Radiology 2008 249 836 844 10.1148/radiol.2493080240 19011184

[B16] Mijnhout GS Hoekstra OS Van Tulder MW Teule GJ Deville WL Systematic review of the diagnostic accuracy of 18F-fluorodeoxyglucose positron emission tomography in melanoma patients Cancer 2001 91 1530 1542 10.1002/1097-0142(20010415)91:8<1530::AID-CNCR1162>3.0.CO;2-# 11301402

[B17] Xing Y Bronstein Y Ross MI Askew RL Lee JE Gershenwald JE Royal R Cormier JN Contemporary diagnostic imaging modalities for the staging and surveillance of melanoma patients: a meta-analysis J Natl Cancer Inst 2011 103 129 142 10.1093/jnci/djq455 21081714 PMC3022618

[B18] Acland KM Healy C Calonje E O’Doherty M Nunan T Page C Higgins E Russell-Jones R Comparison of positron emission tomography scanning and sentinel node biopsy in the detection of micrometastases of primary cutaneous malignant melanoma J Clin Oncol 2001 19 2674 2678 11352959 10.1200/JCO.2001.19.10.2674

[B19] Belhocine T Pierard G De Labrassinne M Lahaye T Rigo P Staging of regional nodes in AJCC stage I and II melanoma: 18FDG PET imaging versus sentinel node detection Oncologist 2002 7 271 278 10.1634/theoncologist.7-4-271 12185291

[B20] Fink AM Holle-Robatsch S Herzog N Mirzaei S Rappersberger K Lilgenau N Jurecka W Steiner A Positron emission tomography is not useful in detecting metastasis in the sentinel lymph node in patients with primary malignant melanoma stage I and II Melanoma Res 2004 14 141 145 10.1097/00008390-200404000-00011 15057045

[B21] Klein M Freedman N Lotem M Marciano R Moshe S Gimon Z Chisin R Contribution of whole body F-18-FDG-PET and lymphoscintigraphy to the assessment of regional and distant metastases in cutaneous malignant melanoma: a pilot study Nuklearmedizin 2000 39 56 61 10834191

[B22] Rinne D Baum RP Hor G Kaufmann R Primary staging and follow-up of high risk melanoma patients with whole-body ^18^F-fluorodeoxyglucose positron emission tomography: results of a prospective study of 100 patients Cancer 1998 82 1664 1671 10.1002/(SICI)1097-0142(19980501)82:9<1664::AID-CNCR11>3.0.CO;2-2 9576286

[B23] Singh B Ezziddin S Palmedo H Reinhardt M Strunk H Tuting T Biersack HJ Ahmadzadehfar H Preoperative ^18^F-FDG-PET/CT imaging and sentinel node biopsy in the detection of regional lymph node metastases in malignant melanoma Melanoma Res 2008 18 346 352 10.1097/CMR.0b013e32830b363b 18781133

[B24] Eigtved A Andersson AP Dahlstrom K Rabol A Jensen M Holm S Sorensen SS Drzewiecki KT Hojgaard L Friberg L Use of fluorine-18 fluorodeoxyglucose positron emission tomography in the detection of silent metastases from malignant melanoma Eur J Nucl Med 2000 27 70 75 10.1007/PL00006666 10654150

[B25] Hafner J Schmid MH Kempf W Burg G Kunzi W Meuli-Simmen C Neff P Meyer V Mihic D Garzoli E Jungius KP Seifert B Dummer R Steinert H Baseline staging in cutaneous malignant melanoma Br J Dermatol 2004 150 677 686 10.1111/j.0007-0963.2004.05870.x 15099363

[B26] Vereecken P Laporte M Petein M Steels E Heenen M Evaluation of extensive initial staging procedure in intermediate/high-risk melanoma patients J Eur Acad Dermatol Venereol 2005 19 66 73 10.1111/j.1468-3083.2004.01130.x 15649194

[B27] Crippa F Leutner M Belli F Gallino F Greco M Pilotti S Cascinelli N Bombardieri E Which kinds of lymph node metastases can FDG PET detect? A clinical study in melanoma J Nucl Med 2000 41 1491 1494 10994727

[B28] Maubec E Lumbroso J Masson F Suciu V Kolb F Mamelle G Cavalcanti A Boitier F Spatz A Aupérin A Leboulleux S Avril MF F-18 fluorodeoxy-D-glucose positron emission tomography scan in the initial evaluation of patients with a primary melanoma thicker than 4 mm Melanoma Res 2007 17 147 154 10.1097/CMR.0b013e32815c10b0 17505260

[B29] Reinhardt MJ Kensy J Frohmann JP Willkomm P Reinhold U Grunwald F Biersack HJ Bender H Value of tumour marker S-100B in melanoma patients: a comparison to ^18^F-FDG PET and clinical data Nuklearmedizin 2002 41 143 147 12109034

[B30] Delgado-Bolton RC Fernandez-Perez C Gonzalez-Mate A Carreras JL Meta-analysis of the performance of ^18^F-FDG PET in primary tumor detection in unknown primary tumors J Nucl Med 2003 44 1301 1314 12902422

[B31] Gould MK Maclean CC Kuschner WG Rydzak CE Owens DK Accuracy of positron emission tomography for diagnosis of pulmonary nodules and mass lesions: a meta-analysis JAMA 2001 285 914 924 10.1001/jama.285.7.914 11180735

[B32] Huebner RH Park KC Shepherd JE Schwimmer J Czernin J Phelps ME Gambhir SS A meta-analysis of the literature for whole-body FDG PET detection of recurrent colorectal cancer J Nucl Med 2000 41 1177 1189 10914907

[B33] Schwimmer J Essner R Patel A Jahan SA Shepherd JE Park K Phelps ME Czernin J Gambhir SS A review of the literature for whole-body FDG PET in the management of patients with melanoma Q J Nucl Med 2000 44 153 167 10967625

[B34] Aukema TS Valdes Olmos RA Wouters MWJM Klop WMC Kroon BBR Vogel WV Nieweg OE Utility of preoperative ^18^F-FDG PET/CT and brain MRI in melanoma patients with palpable Lymph node metastases Ann Surg Oncol 2010 17 2773 2778 10.1245/s10434-010-1088-y 20422454

[B35] Bastiaannet E Wobbes T Hoekstra OS Van der Jagt EJ Brouwers AH Koelemij R De Klerk JM Oyen WJ Meijer S Hoekstra HJ Prospective comparison of [^18^F]fluorodeoxyglucose positron emission tomography and computed tomography in patients with melanoma with palpable lymph node metastases: diagnostic accuracy and impact on treatment J Clin Oncol 2009 27 4774 4780 10.1200/JCO.2008.20.1822 19720925

[B36] Strobel K Dummer R Husarik DB Perez Lago M Hany TF Steinert HC High-risk melanoma: accuracy of FDG PET/CT with added CT morphologic information for detection of metastases Radiology 2007 244 566 574 10.1148/radiol.2442061099 17641374

[B37] Veit-Haibach P Vogt FM Jablonka R Kuehl H Bockisch A Beyer T Dahmen G Rosenbaum S Antoch G Diagnostic accuracy of contrast-enhanced FDG-PET/CT in primary staging of cutaneous malignant melanoma Eur J Nucl Med Mol Imaging 2009 36 910 918 10.1007/s00259-008-1049-x 19156409

[B38] Brady MS Akhurst T Spanknebel K Hilton S Gonen M Patel A Larson S Utility of preoperative [18]F fluorodeoxyglucose-positron emission tomography scanning in high-risk melanoma patients Ann Surg Oncol 2006 13 525 532 10.1245/ASO.2006.02.008 16474909

[B39] Fryback DG Thornbury JR The efficacy of diagnostic imaging Med Decis Making 1991 11 88 94 10.1177/0272989X9101100203 1907710

[B40] Verfahrensordnung des Gemeinsamen Bundesausschusses http://www.g-ba.de/downloads/62-492-634/VerfO_2012-06-21.pdf

[B41] Köbberling J Trampisch HJ Windeler J Memorandum for the evaluation of diagnostic measures Clin Chem Clin Biochem 1990 28 873 879 2081960

[B42] Updating QUADAS: Evidence to Inform the Development of QUADAS-2 http://www.bris.ac.uk/quadas/resources/quadas2reportv4.pdf

[B43] Altman DG Bland JM Statistics notes: units of analysis BMJ 1997 314 1874 10.1136/bmj.314.7098.1874 9224131 PMC2127005

[B44] Kokoska MS Olson G Kelemen PR Fosko S Dunphy F Lowe VJ Stack BC Jr The use of lymphoscintigraphy and PET in the management of head and neck melanoma Otolaryngol Head Neck Surg 2001 125 213 220 10.1067/mhn.2001.118181 11555756

[B45] Longo MI Lazaro P Bueno C Carreras JL Montz R Fluorodeoxyglucose-positron emission tomography imaging versus sentinel node biopsy in the primary staging of melanoma patients Dermatol Surg 2003 29 245 248 10.1046/j.1524-4725.2003.29058.x 12614417

